# Exploring experiences of gratitude during musicking in a community orchestra: an intrinsic case study

**DOI:** 10.3389/fpsyg.2026.1717657

**Published:** 2026-03-06

**Authors:** Liesl Van der Merwe, Debra Joubert

**Affiliations:** Faculty of Humanities, North-West University, Potchefstroom, South Africa

**Keywords:** appreciation, community orchestra, gratitude, positive emotions, reciprocity

## Abstract

**Introduction:**

Within the context of growing scholarly interest in the role of positive emotions and virtues in music education, this study explores experiences of gratitude during musicking among members of diverse cultures and ages (10–79) in a community orchestra in South Africa.

**Methods:**

A qualitative intrinsic case study design was employed to explore 13 orchestra members’ experiences of gratitude while musicking, using open-ended, semi-structured interviews. The data was thematically analysed using ATLAS.ti 25.

**Results:**

Three central themes were generated from the data analysis: (1) gratitude as experienced across ages (10–79) and cultures within the orchestra, (2) the triadic view of gratitude, encompassing benevolent intentions, benefits received, and the roles of beneficiaries and benefactors within the orchestra community, and (3) three upward cycles—the chain of appreciation, the chain of reciprocity, and the broaden-and-build features of gratitude—that foster social cohesion, prosocial behaviour, individual well-being, and better musical performances.

**Discussion:**

The study demonstrated that understanding appreciation and reciprocity chains can encourage gratitude, which in turn broadens musical and interpersonal skills and builds flourishing individuals and music communities.

## 1 Introduction

This study is about the experiences of gratitude during musicking in a community orchestra. Small (1998) defines musicking as a participatory and relational practice in which musical meaning is generated through embodied interactions among participants during a performance. In an orchestra, members are interdependent and must collaborate. Orchestra members share responsibility for the quality of performances. Within this relational context, gratitude emerges through ensemble participation, which enhances musicians’ awareness of, reliance on, and appreciation for one another.

Gratitude can influence the experiences and outcomes within a community orchestra since gratitude is a significant internal catalyst, prompting individuals to acknowledge and respond positively to perceived acts of kindness from others (Allen, 2018; Yoshimura and Berzins, 2017). Gratitude confirms and maintains social ties and sets a chain of reciprocity in motion. Therefore, gratitude is essential in creating social cohesion and community (Komter, 2004).

Gratitude is both an emotion and a virtue. It has also been described as an action, an expression of gratitude, that follows the realisation, acknowledgement, and appreciation that someone has been undeservingly (Emmons, 2004) kind to you (Gulliford et al., 2013). Fredrickson (2004) states that “gratitude arises when an individual (beneficiary) perceives that another person (benefactor) or source (e.g., God, luck, fate, in this case, the orchestra) has intentionally acted to improve the beneficiary’s well-being” (p. 150). This definition implies a triadic concept consisting of the benefit/gift, benefactor/giver and beneficiary/receiver (Rusk et al., 2016).

Roberts (2004) adds that the giver’s intention should be benevolent and that the receiver should construe the giver’s intention as good. The good intentions have important implications for music education and community music. Our teaching and musicking must be explicitly intended for the benefit of the orchestra members and community members. Fostering a sense of gratitude for participating in a community orchestra can teach orchestra members to recognise and express gratitude towards their fellow musicians, mentors, and audiences. This, in turn, promotes a supportive and collaborative musical environment (Buck, 2004).

Previous research has highlighted the fundamental role of gratitude in education. Studies by Jin and Wang (2019) and Howells (2012) have shown how embracing gratitude can have a transformative influence on fostering positive learning environments and improving educational outcomes. Practising gratitude in educational settings leads to a more positive attitude towards learning, such as enhanced focus, effort and resilience (Caleon et al., 2019; Wilson, 2016). There is a positive correlation between gratitude and academic motivation and achievement, resulting in numerous positive learning-related outcomes (Jin and Wang, 2019; King et al., 2023; Valdez et al., 2022).

Although the concept of gratitude has been widely researched in psychology and education, often through themes such as moral cohesion, prosociality, humility, recognition, emotional connection and well-being (Allen, 2018; Gulliford and Morgan, 2021; Howells, 2012; Roberts and Telech, 2019), its specific implications within the context of music education and community music remain relatively unknown (Bernabé-Valero et al., 2019). Higgins (2012) states that the music facilitator engages in musicking through the act of giving time, space, quality experiences and skills. However, he warns that the gesture of gift-giving might come with indebtedness and expectations of reciprocity. On the other hand, Ascenso (2016) states that musicking in community settings contributes to musicians’ well-being by generating positive emotions such as gratitude. These different views on gratitude need to be further explored in the context of a community orchestra.

The John Templeton Foundation commissioned a white paper to report on the state of the science of gratitude. Allen (2018) writes: “Still much is unknown about how children of different ages experience and develop gratitude.” “Similarly, there is still considerable room for future studies to examine cultural differences in gratitude experiences” (p. 57). This study was motivated by the lack of gratitude studies in music education and community music and the gaps identified in the Templeton Foundation white paper.

By exploring gratitude within a community orchestra, this study advances theoretical understandings of gratitude by conceptualising it as a relational process developed through collective engagement rather than a response to isolated acts of kindness. Therefore, the purpose of this qualitative intrinsic case study is to explore experiences of gratitude during musicking among members of different cultures and ages (10–79) in a community orchestra in South Africa. The research question that guided this intrinsic case study was: What is the nature of gratitude experiences during musicking among members of different cultures and ages (10–79) in a community orchestra in South Africa?

## 2 Procedures

### 2.1 Research design

The research approach of this study is a qualitative intrinsic case study with an interpretivist worldview. According to Merriam and Tisdell (2015) the unit of analysis characterises a case study. Similarly, Creswell and Poth (2018) emphasise that case studies are bounded by time, place and activity. In this study, the bounded case is a single community orchestra in South Africa. The community orchestra, which started as a youth orchestra, rehearses every Friday afternoon during university semesters from 15:00 to 18:00 at a tertiary institution in South Africa.

### 2.2 Participants

All active members of the community orchestra were invited, through the orchestra WhatsApp group, to participate in this study. 13 Orchestra members volunteered to participate in the study, and each chose their own pseudonym. In 2024, at the time of data collection, the orchestra consisted of 48 members, with ages ranging from 10 to 79 years old. The members include school children, university students, and adults. The inclusion of children (6–12 years old), adolescents (13–18 years old), university students, adults, and people over 65 years of age in the research study was based on the aim of exploring a range of experiences across different age groups within the community orchestra (Table 1). Each age group offers unique insights into experiences of gratitude and musicking, contributing to a holistic understanding of the phenomenon under investigation. Members speaking different languages (Afrikaans, English, Setswana, XiTsonga, Sesotho, Sepedi) participated in the study, further contributing to the sample’s diversity (Table 1).

**Table 1:** A demographic table of the participants.

Pseudonym	Language	Age	Developmental stage
Jeff	Afrikaans	10	Middle childhood
Pirate	Afrikaans	13	Adolescence
Petrus	Afrikaans	16	
123NieEkNie	Afrikaans	17	
Rose	Afrikaans	18	
Sebopa	Sepedi	19	Early adulthood
Teddy	XiTsonga	23	
Mandoza	Sesotho	24	
Jack Sparrow	Setswana	28	
Zorro	Afrikaans	36	Middle age
Bella	Afrikaans	41	
Elise	Afrikaans	49	
Falcoman	English	79	Late adulthood

Each developmental stage is indicated by a distinct color to provide a visual grouping according to the participants’ age groups.

### 2.3 Data collection

Data were collected through individual, open-ended, semi-structured interviews lasting 30 to 45 min. No follow-up interviews were necessary. Participants could choose the time and place of their interviews, in person or via Zoom, and they all signed informed consent forms. The interviews were conducted in the participants’ preferred languages: Afrikaans for Afrikaans-speaking participants and English for English- and African-language-speaking participants. Under-aged children were asked for their assent and parental or guardian consent before the interviews. Adult participants gave informed consent. Participation was voluntary.

To mitigate potential power imbalances, all interviews were conducted by an independent person, Debra Joubert, who had no leadership, teaching, or evaluative role within the orchestra. All recruitment and interview-related communication occurred directly between her and the participants. Participant anonymity was safeguarded through a clear separation of researcher roles and restricted access to data. Identifying information and personal details were not disclosed to the conductor (the first author) and only participant pseudonyms were shared with her. The conductor was not involved in recruiting participants or collecting data and did not have access to identifying information or raw interview recordings. She only had access to the transcribed interviews. Participants were informed of these arrangements prior to participation.

Interview questions focused on participants’ experiences of gratitude during musicking in the community orchestra. The interview questions were organised into three broad areas: participants’ musical involvement and relationships within the orchestra; participants’ general understandings and cultural experiences of gratitude; and their specific experiences of gratitude during orchestra rehearsals and performances. Questions about musical participation and interpersonal relationships within the orchestra prompted open-ended responses that encouraged participants to share personal and relational experiences of gratitude (see Supplementary material for the detailed interview questions, which were adapted to accommodate different ages).

### 2.4 Data analysis

As part of the analysis process, the first author included all the transcribed interviews in one heuristic unit in ATLAS.ti 25. Data analysis employed thematic analysis (Braun and Clarke, 2021) utilising ATLAS.ti 25 software. This process involved systematically coding and categorising the collected data to identify recurring patterns and themes related to gratitude within the context of the community orchestra. The analysed data consisted of 13 documents, 228 codes and 492 quotations.

In preparation for this study, we conducted a concept analysis on gratitude across literature in positive psychology, general education and music education. The collected literature data were analysed using ATLAS.ti 25 (Friese, 2019). Our analysis commenced with a deductive approach for the current study, applying a set of *a priori* codes in ATLAS.ti 25 from our concept analysis of gratitude, namely the antecedents of gratitude, the attributes of gratitude, and the consequences of gratitude.

Additionally, an inductive approach was employed to allow for the emergence of new codes from the interview data. The emerging codes, categories, and themes offered valuable insights into the defining attributes of gratitude, thereby enhancing the conceptual clarity surrounding gratitude within the context of music education and community music.

## 3 Findings

**Figure 1:**
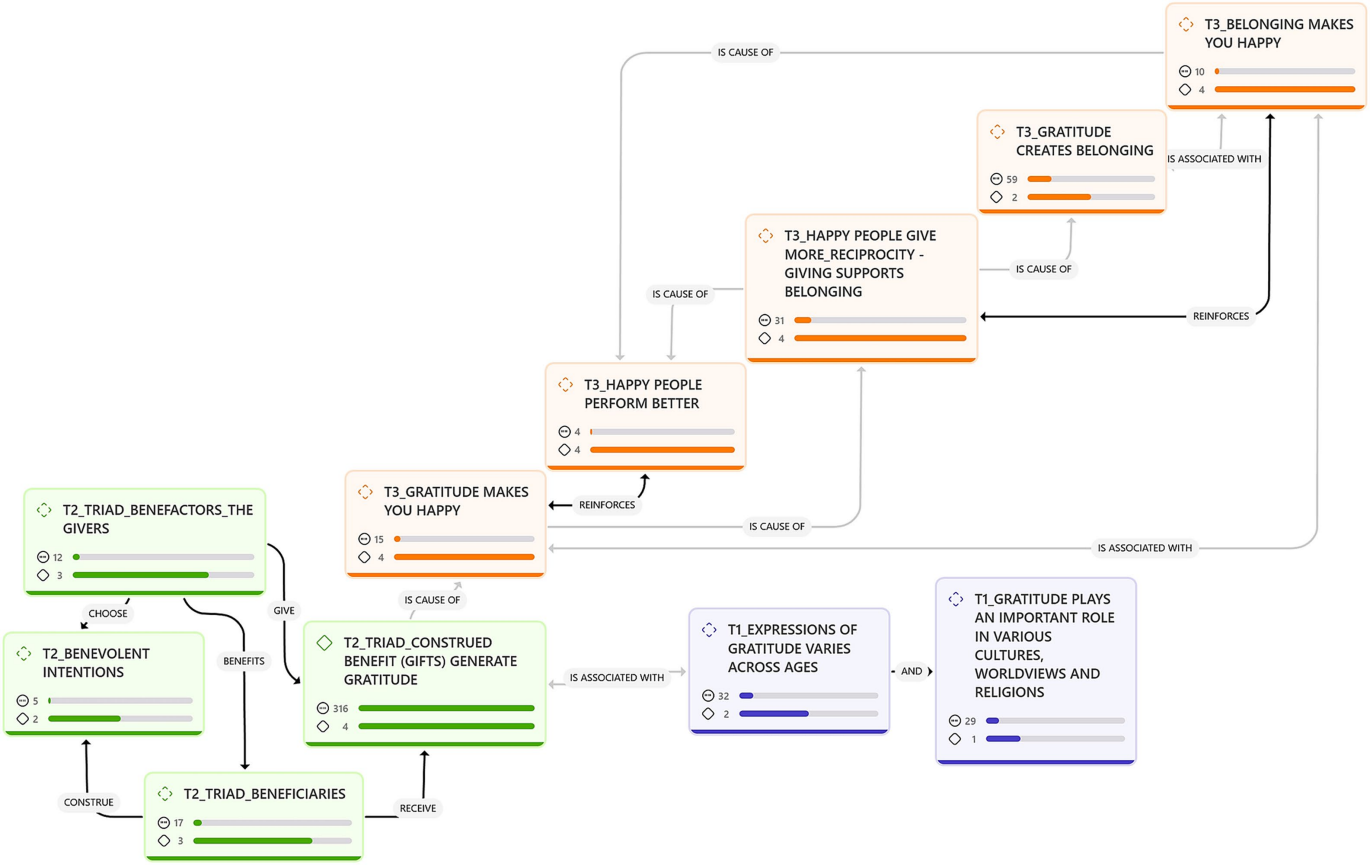
An ATLAS.ti 25 network view of the categories across the three themes: how gratitude works in a community orchestra.

In our findings, we unpack three themes: Theme 1 explores gratitude across ages and cultures within the orchestra. In theme 2, we discuss what the data revealed about the triadic view of gratitude, namely, benevolent intentions, the benefits experienced by participants, the beneficiaries within the orchestra, and the benefactors in the community orchestra. Lastly, in theme 3, we examine three upward cycles: the chain of appreciation, the chain of reciprocity, and the broaden-and-build properties of gratitude in the community orchestra. The numbers below each category, in Figure 1, indicate the category’s groundedness and density. Groundedness is the number of quotes in the category, and density indicates the number of links to other categories. The category with the highest groundedness, 316 quotes, is the benefits that the participants are grateful for in the community orchestra.

### 3.1 Theme 1: gratitude across ages and cultures

#### 3.1.1 Gratitude across ages

From the data, it became apparent that younger members and older members express and experience gratitude differently. Teenagers feel that gratitude requires vulnerability (Petrus, 8:41). And in the Gen Z generation they tease each other when they say thank you too often (Pirate, 9:34). Children are more emotional (Falcoman, 4:37) and excited (Zorro, 13:27) in their expression of gratitude and some older adults are more inhibited (Zorro, 13:28) and feel embarrassed by an over emotional expression of gratitude (Falcoman, 4:39). Some adults are more formal (Falcoman, 4:36), inhibited, and brief (Zorro, 13:28) in their expression of gratitude. However, since adults see the impact of the orchestra over the years, they are more grateful (Elise, 3:60). Students repeatedly want to hear that they are appreciated; “they want to be thanked more” (Jack Sparrow, 5:29, Pirate, 9:31). Jack Sparrow says that “the different ages make the orchestra feel like a family” (5:4).

#### 3.1.2 Gratitude across cultures

All the orchestra members were brought up to be grateful. Gratitude is vital in all the different cultures in the orchestra. Some Afrikaners are brought up to appreciate the little things in life (Elise, 3:39) and live joyfully (Bella, 2:32). Afrikaners say thank you often (123Notme, 1:16) and might be more inhibited (Bella, 2:51) in the expression of gratitude. Still, it is essential in their culture to be grateful in all circumstances since it is a Christian principle. Gratitude is expressed in prayer (Rose, 10:11). Jack Sparrow, a Setswana member, says they, too, always pray “At my grandmother’s house, we always pray first” (5:17).

Mandoza says, in the Sesotho culture, “gratitude plays a big role, … you have to acknowledge those people who supported you. So, you have to give them their flowers while they are still alive”. Sebopa is Sepedi, who says you must “do good things for others even though you don’t know them”.

In the XiTsonga, Sepedi, Setswana, and Sesotho cultures, they have big cultural gatherings twice a year where there are ceremonies of slaughtering a cow, “and then they stay all night together, singing and praying” (Jack Sparrow, 5:33). They call this the feast (Mandoza, 7:16). Sebopa says, “it’s a gratitude ceremony because the whole purpose of the ceremony is to bring people together and get to know each other” (11:24). Mandoza also says this is a gratitude ceremony because “we normally do the ceremonies to express gratitude to the person who you want to acknowledge” (7:15).

### 3.2 Theme 2: a triadic view of gratitude

**Figure 2:**
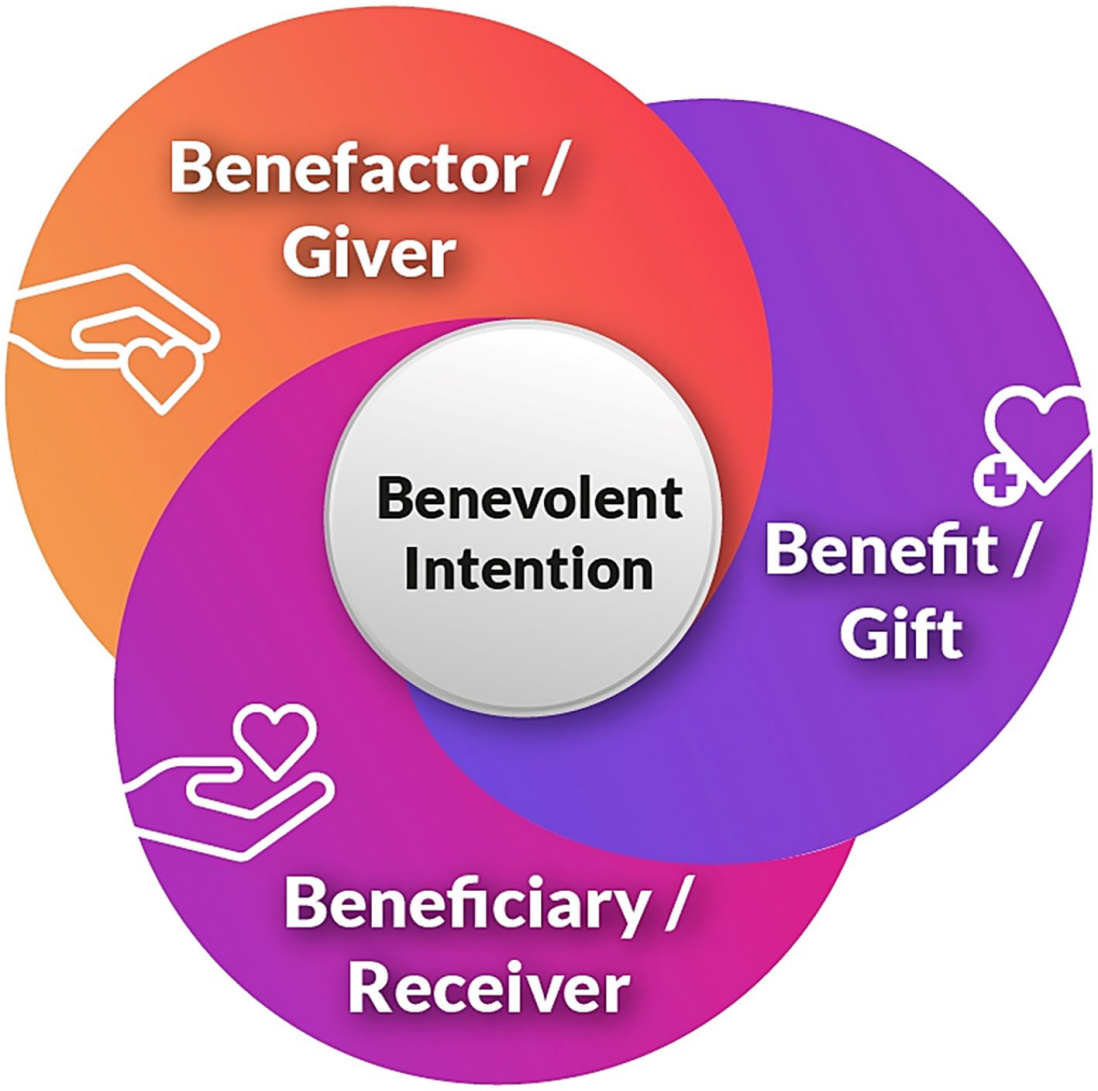
A triadic view of gratitude in a community orchestra.

Since gratitude implies a concept consisting of benevolent intentions, benefits, benefactors, and beneficiaries (Figure 2), we analysed the data to understand these aspects of the gratitude system better.

#### 3.2.1 Triadic view: benevolent intentions

Before a concert, one of the conductors, Liesl van der Merwe, often asks the orchestra, “What are our intentions for this concert tonight?” They say things like “sharing love and compassion through music, to provide an escape for the audience, healing, to enjoy making music together and to feel proud of our accomplishments” (3:7). Teddy says, “When we’re with the orchestra, we’re doing something we love” (12:24). … “we’re all here to do one thing. We’re all here to learn. We’re all here to make music” (12:25).

#### 3.2.2 Triadic view: appreciate benefits

In the data analysis, the theme of appreciating benefits had 316 quotes, which is by far the most grounded category. The benefits that participants appreciated included: the beauty of the orchestral sound, the kindness of all the stakeholders, the sharing of gifts, a sense of support, that making music as a team fosters closeness, the cultivation of good relationships, the opportunities the orchestra afforded, individual and collective accomplishments, enhanced well-being, and profound spiritual experiences.

##### 3.2.2.1 Appreciate the beauty of the orchestral sound

Interestingly, when the participants talk about what they love about the orchestra they speak about the sound of the orchestra: 123Notme says, “My favourite part is when all the bass instruments play one loud long strong note” (1:5). Similarly, Rose says “to produce such a broad sound is unbelievable” (10:5). Mandoza says it was the sound of the orchestra that motivated him to play (7:3). Bella says, “Every time I hear the merging of the sounds, then I am grateful” (2:35).

##### 3.2.2.2 Appreciate kindness

Many musicians mentioned feeling comfortable in the orchestra because everyone is kind. Jack Sparrow says, “I’m grateful for the way they [the conductors] show us how to play. They don’t shout at us. They show us in a nice way” (5:20). The musicians are also kind to each other. Jeff appreciates that the orchestra members showed empathy when he got hurt (6:28). Kindness is also sometimes shown by giving gifts.

##### 3.2.2.3 Appreciate gifts

123Notme tells the story of when one of the orchestra members became a father, and many members brought gifts for the baby (1:23). Pirate, a young member of the orchestra, once brought all her Easter eggs to the orchestra rehearsal and told everyone “I am giving Easter eggs to all my favourite members” (9:39), which was everyone. Elise appreciates the parents’ hospitality in bringing food and presenting it with so much love during our breaks and camps (3:49).

##### 3.2.2.4 Appreciate feeling supported

The musicians experience the orchestra as a nonjudgmental space where they are helped rather than criticised when making mistakes. They feel safe to make mistakes. Falcoman mentions, “If somebody makes a mistake, it doesn’t bother you. You work on that together” (4:32). Furthermore, they appreciate being helped by their desk partners. Mandoza says we work together, and we help each other when someone needs help (7:4). Similarly, Sebopa says, “In our section, we’ve got full support because if you struggle with something, your members are able to help you, regardless of whether you are friends or not” (11:15). The musicians feel thrilled when they receive compliments from each other, the conductor or the audience after a good performance. Petrus explains, “You always question yourself afterwards. So, then it is nice when people motivate or encourage you and says that was fantastic, don’t worry” (8:23). Petrus says another gesture he appreciates is the stamping of feet that communicates “you’ve really played beautiful. Or after struggling with a difficult part, you’ve now mastered it. We are proud of you” (8:29).

A helping ritual that everyone mentioned was working as a team to put out the chairs and music stands before a rehearsal and packing them away afterwards. An older member, Falcoman, when he was ill, especially appreciated it when ladies helped him carry his instruments up the stairs (4:24). Helping each other brings people together, but also brings music together. All the participants appreciated that their family and friends supported their orchestra participation, and they talked with great excitement about their churches (Mandoza, 7:10; Sebopa, 11:19) and brilliant music teachers (123Notme, 1:14; Falcoman, 4:19; Rose, 10:12) who supported their musical development.

##### 3.2.2.5 Making music as a team brings us closer together

Petrus sums it up beautifully “If you are musicians together and you make music together, then it brings you closer together because you identify with each other” (8:21). Pirate says, “To play music for an hour with your favourite people is almost therapeutic” (9:38). Similarly, Sebopa says “the music brings us together, … regardless of where you come from and what you have and what you don’t have” (11:10).

##### 3.2.2.6 Appreciate good relationships, friendships and social bonds

All the musicians are deeply grateful for their meaningful relationships with each other in the orchestra. They speak of loving relationships because they feel like their friends understand them. There are respectful relationships with older members and between conductors; there are even bonds between musicians who hardly know each other and come from entirely different backgrounds and cultures. Many children made friends in the orchestra, with whom they are also friends at school. Jeff says he would see someone from the orchestra at school, “and then we greet each other and give each other a hug” (6:29). Petrus says, “I would have had fewer friends if it were not for the orchestra” (8:7). Similarly, Teddy realised that she started talking to more people, “people I wouldn’t usually hang out with” (12:27).

##### 3.2.2.7 The orchestra opened doors for me

Mandoza said, “The orchestra opened doors for me” (7:19). The musicians are very grateful for various opportunities the orchestra afforded them, namely:

- to learn to read music (Mandoza, 7:12; Sebopa, 11:1);
- to have an instrument (Pirate, 9:13);
- to be in the orchestra (123Notme, 1:12; Bella, 2:6);
- for a rehearsal space (Bella, 2:46);
- learning about the music from the conductors (Jack Sparrow, 5:5);
- becoming better musicians (Sebopa, 11:8);
- for the opportunity to learn valuable transferable skills in the orchestra (Sebopa, 11:36), and;
- the orchestra led to opportunities in other contexts (Falcoman, 4:27; Sebopa, 11:4).

##### 3.2.2.8 Experienced individual and communal accomplishments

Musicians appreciate being given a solo, good concerts and celebrating individual and communal successes. Mandoza says, “Being given a solo is one of the best moments I’ve ever experienced” (7:20). Petrus says he is “happy to get the opportunity to sing a solo with the orchestra at concerts” (8:1). Pirate tells of one time her “friends were at one of the orchestra concerts and after the last note was played they stood up and clapped enthusiastically with big smiles”. This made her very happy that they enjoyed the concert. Successes are also celebrated during rehearsals between desk partners (Teddy, 12:20) and when the whole orchestra stamp their feet in appreciation (Petrus, 8:29).

##### 3.2.2.9 Well-being

Orchestra members said playing in the orchestra is good for their physical and mental health. Elise says being in the orchestra helps with “the feeling of purpose in your life. And if you have a purpose, then it feels like your life makes sense, and that improves your well-being” (3:62). Elise has also sometimes felt physically unwell before a rehearsal, and afterwards she would feel well again. Falcoman says, “Sometimes you don’t feel like it, but when you’ve finished, you’ve been elevated. Similarly, Bella experiences playing in the orchestra as “a very uplifting experience” (2:3). It is “lifting her spirit” (2:52) and after rehearsals she feels “restored” (2:56). Sebopa goes into a rehearsal with academic stress, and he says “just being there and interacting with each other made me feel at ease and gave me hope that everything is going to be all right” (11:40).

##### 3.2.2.10 Spirituality

Orchestra members appreciate having had spiritual experiences, such as flow experiences, awe and wonder, a thrill of being the driving force (Petrus, 8:13), and godly synchrony, as Falcoman puts it, “being together is next to godliness” (4:24). Bella has experienced overwhelming gratitude for being part of something bigger than herself (2:4). Elise experiences cosmic gratitude for all that is good and in harmony in the universe. She explains, “The harmony in the orchestra symbolises everything going right in the world. I am grateful for all of it, for all that is good, and beautiful, and in harmony, for everything that is together and full of love” (3:47). Elise is grateful to God that she can make music (3:34). Bella has been in awe and wonder of the conductors and the musicians’ talent and passion (2:17, 2:19, 2:41).

#### 3.2.3 Triadic view: benefactors

Jeff, one of the young players, made the profound statement that “every single person plays a significant role with the people around them” (6:27). Similarly, Rose says, “Everyone has a role in the orchestra, and I am grateful that they are there to fulfil their role” (10:17). So, each orchestra member is a benefactor. Bella is “grateful for the people who are at the rehearsal, and that they can be there and give what they can give” (2:43). For some, it is a sacrifice to be there because they have to juggle family life and playing in the orchestra (Bella, 2:13).

Teddy and Pirate are benefactors of positive energy. Pirate says, “I’ve seen at the orchestra when I am excited, then it also makes the other members happy. … I think it is a contagious smile” (9:24).

An interesting observation made by Elise is that “the giver is in a privileged position to give, and the receiver sometimes does not choose not to be able to give. So, there is a power imbalance. The giver is in a stronger position, and the receiver is in a weaker position” (2:45). She was referring to the people who volunteer to provide food during break times.

Many orchestra members are Christians and see God as the ultimate giver and the source of their gratitude. As Bella says, “I have a very personal relationship with God, and He is my absolute source of gratitude. Even just the fact that I can hear and have the talent to make music, and for the opportunities I had to learn an instrument” (2:28). Elise also explained that “Christians are grateful in all circumstances, also in difficult circumstances” (3:40).

#### 3.2.4 Triadic view: beneficiaries

The beneficiaries are the orchestra and their audiences. To experience gratitude, the beneficiaries must construe the conductors’ and musicians’ intentions as kind, which they do. Rose says, “I am still new in the orchestra, but everyone is very friendly and helpful” (10:8). Mandoza says, “Our conductors make everyone feel comfortable, and that’s what I like about the orchestra” (7:5). Jeff enjoys the performances because of “all the people sitting there and listening to our music”(6:6). He enjoys seeing other people happy.

### 3.3 Theme 3: three upward cycles: appreciation, reciprocity and broaden-and-build

**Figure 3:**
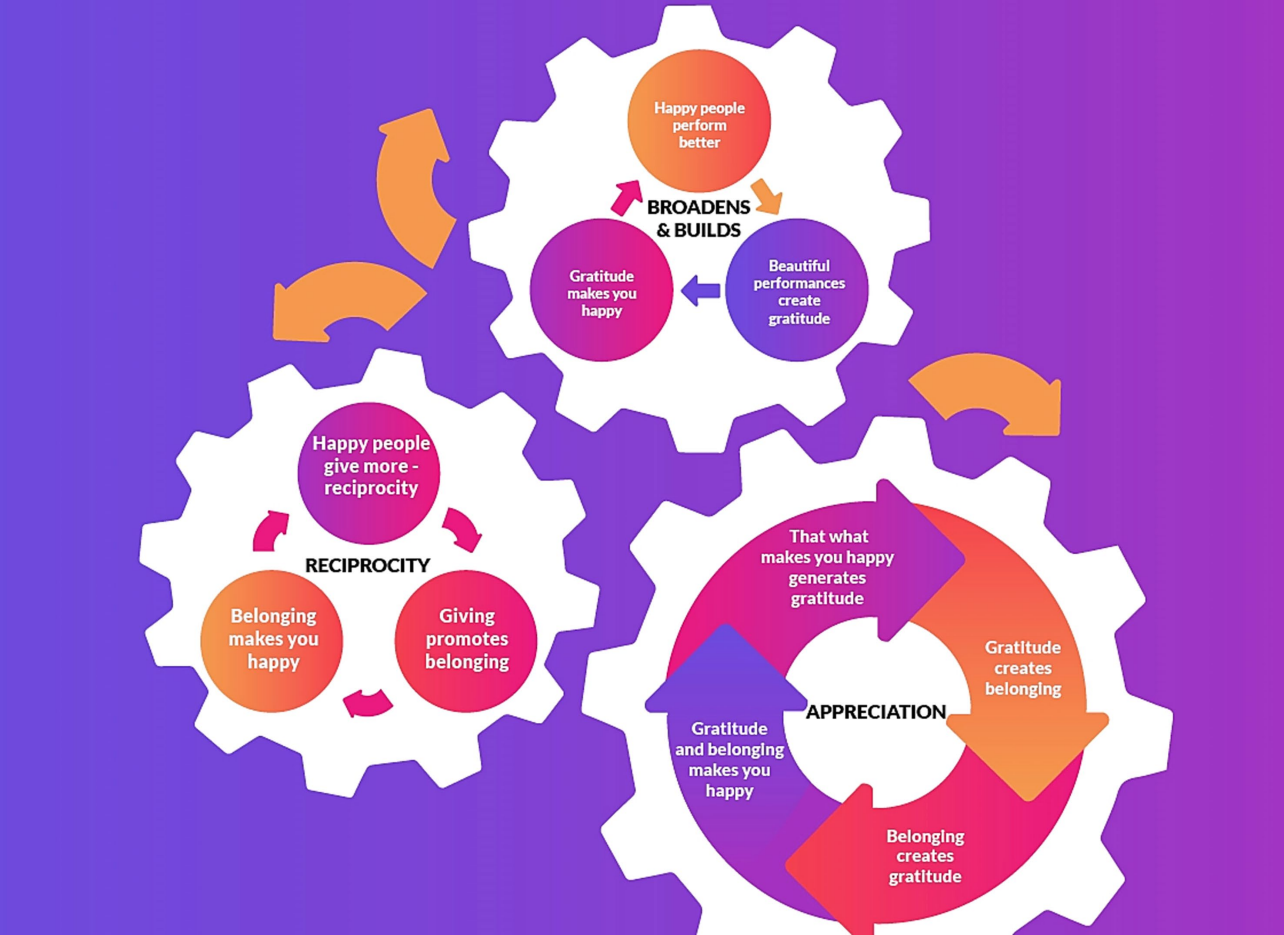
Three upward cycles of gratitude: appreciation, reciprocity, and broaden-and-build.

From the data, it became clear that there are iterative cycles (Figure 3) at work in the musicians’ gratitude experiences. The data revealed strong similarities across age groups and cultural backgrounds in experiences of gratitude, happiness and belonging. The older participants had a heightened awareness and appreciation of how their experience could enhance the orchestra’s musical quality.

**Figure 4:**
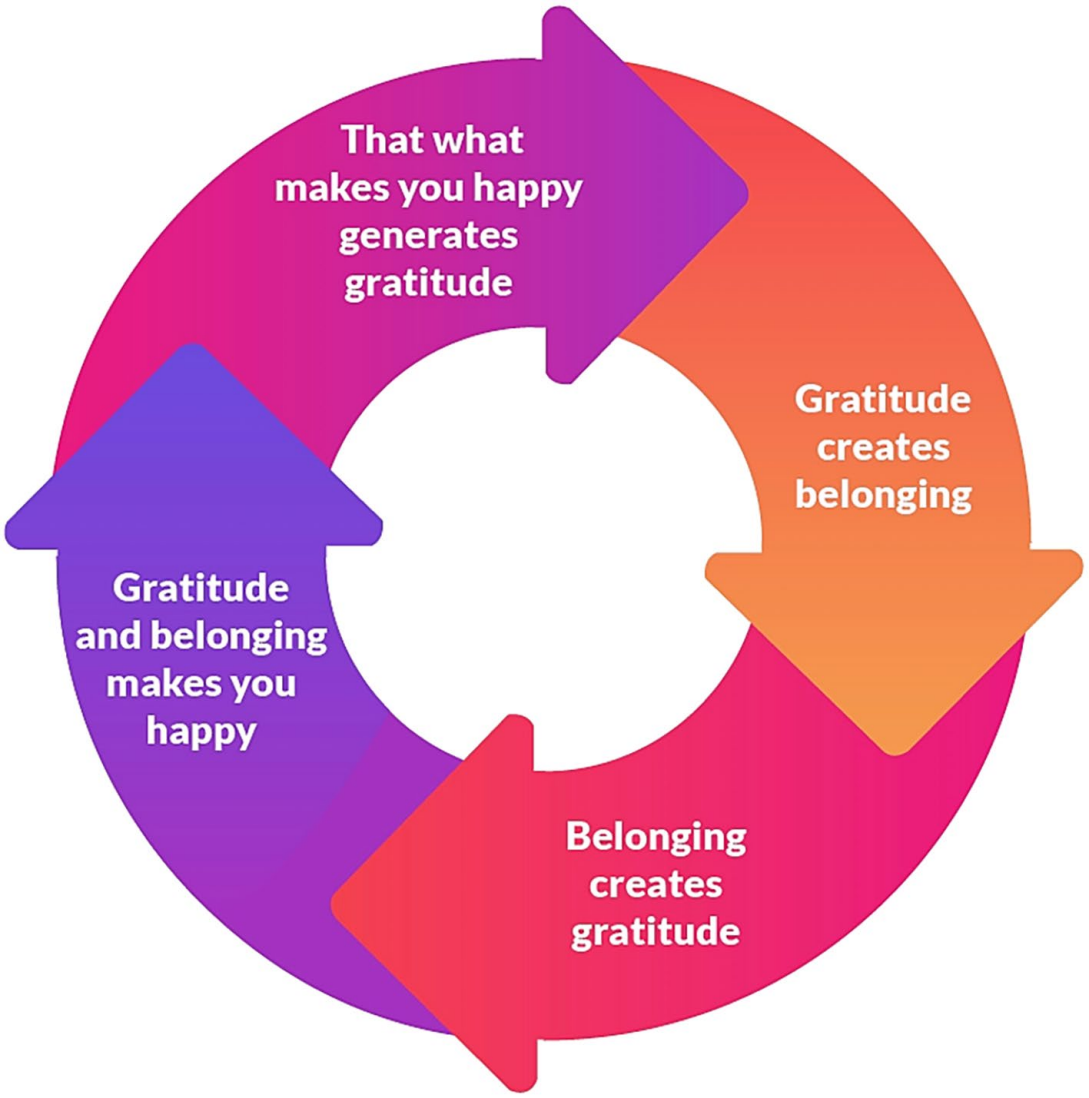
Chain of appreciation in a community orchestra.

**Figure 5:**
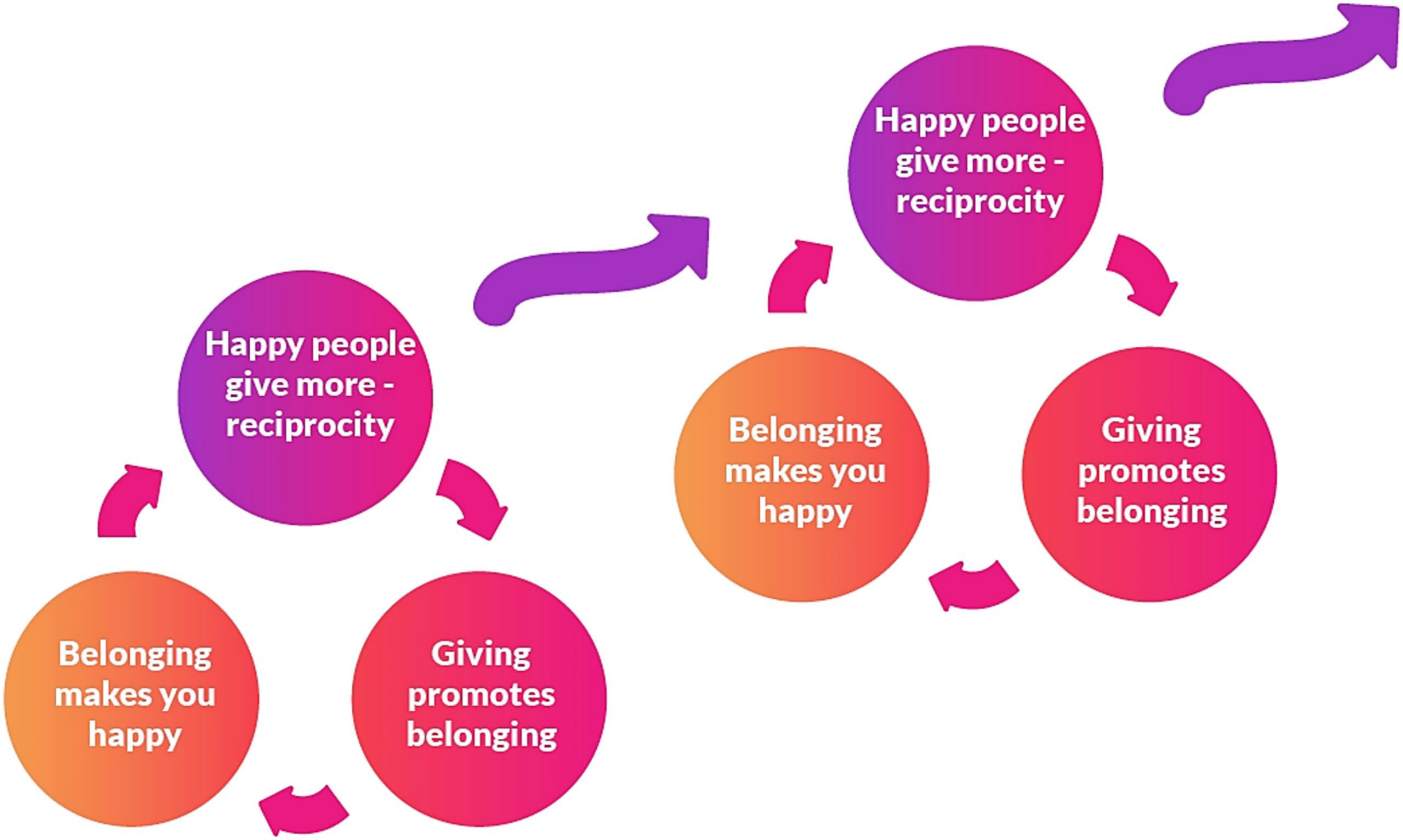
Chain of reciprocity in a community orchestra.

For the past 3 years, the first author has started each rehearsal by asking: “What are you grateful for that happened this week? What are you looking forward to? What can we celebrate together? This gratitude ritual fosters positive emotions and gratitude among members, making them feel a sense of belonging. Belonging, in turn, creates gratitude. Both gratitude and belonging make them happy (Figure 4), and happy people reciprocate more (Figure 5). They perform better when they have experienced positive emotions. I tell them it is like Peter Pan; we need happy thoughts to fly. When you have positive emotions and then play better, it generates further gratitude for the beautiful music. And as Pirate explained so astutely, “That what makes me happy makes me grateful.”

**Figure 6:**
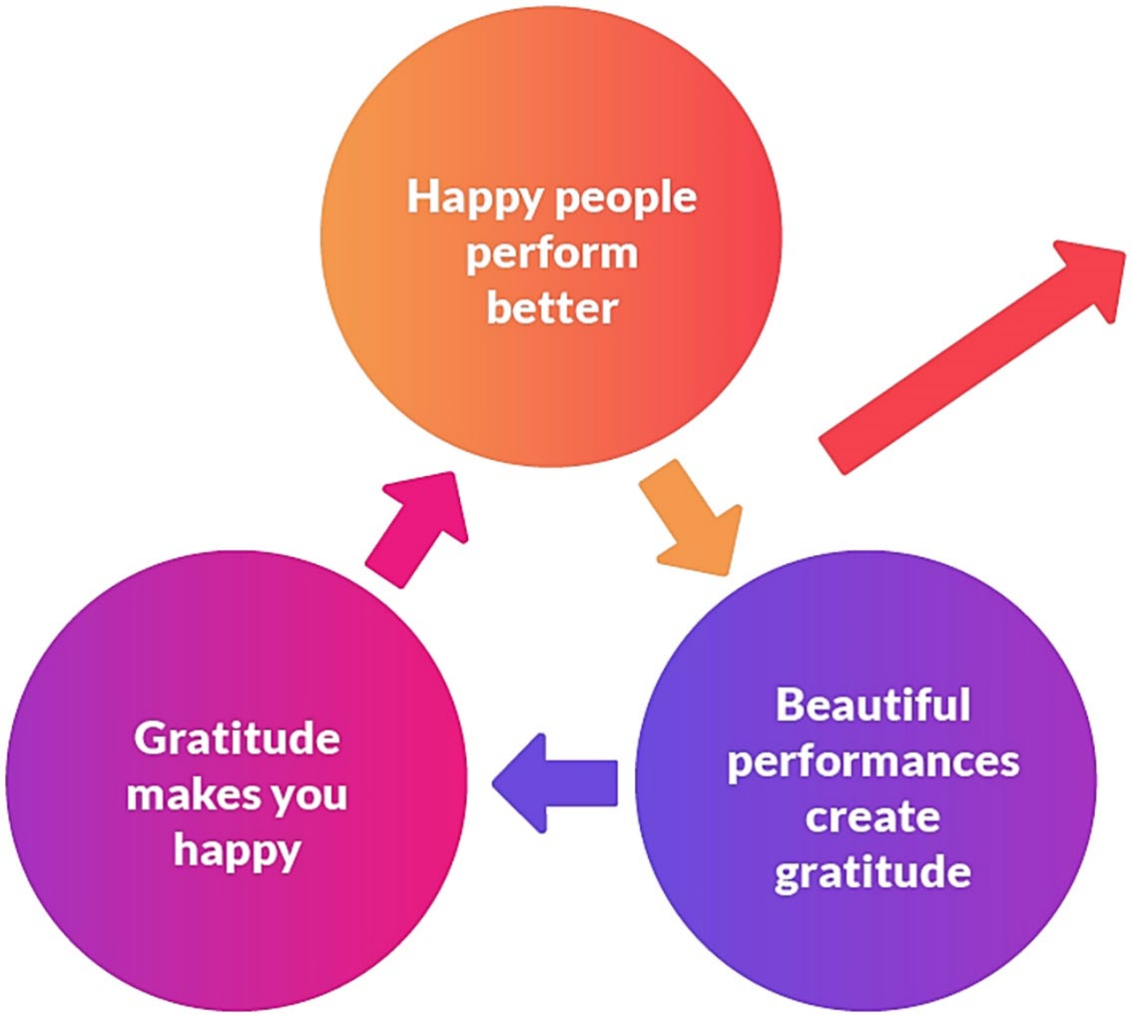
Gratitude broadens and builds skills in a community orchestra.

These upward chains of reciprocity and appreciation are supported in the literature. Fredrickson (2004) explains that, like other positive emotions, gratitude also broadens perceptions and builds resources (Figure 6). The thought-action tendency of gratitude is the urge to behave prosocially towards the benefactor or pay it forward to others. It is an upward spiral since the grateful individual creatively considers prosocial action to express their gratitude (Fredrickson, 2004).

The three iterative cycles (Figure 3)—appreciation (Figure 4), reciprocity (Figure 5), and broaden-and-build (Figure 6)—are illustrated and unpacked in the following discussion.

#### 3.3.1 Cycle one: chain of appreciation

##### 3.3.1.1 Gratitude creates belonging

The gratitude rituals we have in the orchestra are sharing what we are grateful for at the beginning of the rehearsal (123Notme, 1:26), expressing gratitude at the end of the rehearsal and expressing anticipation for the next rehearsal (Elise, 3:13). These rituals create positive interaction ritual chains (Collins, 2014). Other gratitude rituals include celebrating birthdays by playing the happy birthday song and eating cake together (Jeff, 6:25; Zorro, 13:22). Stamping our feet in appreciation when someone plays well (Sebopa, 11:30) is another gratitude ritual. The end-of-year concert is our big gratitude celebration (Zorro, 13:18). We hand out gifts and cards to everyone who made an exceptional contribution, and the conductor thanks each one on stage. All the members who are leaving the orchestra receive a certificate, and the conductor expresses her gratitude for something specific they contributed to the orchestra (Petrus, 8:39). These gratitude rituals create a sense of belonging (Figure 3), which Collins (2014) calls group solidarity. As Sebopa explains, “Every time before a rehearsal, she asks, ‘What stood out for you this week? What was the most amazing thing that you can celebrate that you’ve achieved this week?’ And that helps us to foster cohesion between us and foster social bonds. So those are the moments I enjoy in the orchestra” (11:7).

##### 3.3.1.2 Belonging creates gratitude

Elise says: “I have a strong sense of belonging. When I arrive there on a Friday, I feel wanted. And that is quite something special because it is not at many places where you feel at home and being needed” (3:3). Pirate also appreciates belonging to the orchestra. She says, “When things are tough with friends at school, I always tell myself that I always have the orchestra. When my school friends are angry with me, then my orchestra friends are just happy to see me, and that is very nice” (9:35). This quote shows that belonging makes Pirate happy.

##### 3.3.1.3 Gratitude and belonging make you happy

Zorro explains this chain of gratitude and happiness beautifully, “So if you are grateful, you are more satisfied. And if you are more satisfied then you are happier” (13:31). So, gratitude makes you happy. Similarly, belonging also makes you happy, as Pirate explains, “I am grateful for my orchestra friends because they make it enjoyable for me” (9:15). Rose also describes how belonging makes her happy, “It is nice to see that no matter how old you are, you can still participate, and no matter how young you are, there is still a place for you. It also doesn’t matter at what level you play, there is an opportunity for you” (10:21).

#### 3.3.2 Cycle two: chain of reciprocity

##### 3.3.2.1 Happy people give more

Elise says, “My good experiences in youth orchestras made me want to do this now, pay it forward” (3:4). Similarly, Falcoman tells of a fantastic music teacher he had, “I learned an enormous amount from him, and I try pass that down to whomever I can” (4:16). Mandoza also pays it forward “I make sure that everything that I learn during orchestra rehearsals, I take it outside and show it to other people” (7:28). Gratitude also changes people’s behaviour as 123Notme explains “If you are grateful then you will treat the other person better, to show that you appreciate it” (1:17). Sebopa experiences this, “Because I am in the orchestra, they are more friendly” (11:3).

##### 3.3.2.2 Giving promotes belonging

Elise explains that “Everybody is a giver because they all play their part, and that helps that they belong” (3:57). Zorro is motivated to contribute to the orchestra because “The orchestra provides a safe haven to play the violin, and also because I know I matter here” (13:2). So being able to contribute makes Zorro feel that she belongs.

##### 3.3.2.3 Belonging makes you happy

The motto of our orchestra is *Our happy place*. Pirate sums this up beautifully: “When people like each other, it makes it more enjoyable for them” (9:27). Bella says, “Making music with like-minded people is enjoyable and good for my soul” (2:11). Similarly, Zorro feels accepted. “I feel good to be with people who are like me” (13:11).

#### 3.3.3 Cycle three: gratitude broadens and builds

##### 3.3.3.1 Happy people perform better

At the beginning of each year, we have a camp. Getting to know each other is very important for the ensemble playing in the orchestra. Elise says, “I always feel that the quality of the relationships determines the quality of the musicking” (3:58). Pirate says, “In my experience, happy people do what they like doing, much better” (9:28). She continues to explain that “if we make it nicer for each other and people enjoy the good company and the sound of the orchestra, then it makes it more enjoyable for them and then they play better” (9:29).

##### 3.3.3.2 Beautiful performances create gratitude

Teddy’s favourite part of playing in the orchestra is “the concerts” (12:4). Falcoman’s most positive memories of the orchestra were “when we’ve had good concerts and had standing ovations” (4:7). Pirate enjoys it when we travel back in the bus after a concert and we say “wow, we played extremely beautiful” (9:7).

##### 3.3.3.3 Gratitude makes you happy

Jack Sparrow said, “There was this other time we were playing the song piece called *Happy*, and I could see that everyone was playing happy, everyone was happy to play that song. And it was really nice. I got butterflies in my tummy, so I enjoyed seeing everyone happy in the orchestra” (9:24).

## 4 Discussion and conclusion

This study illustrated mainly a triadic view of gratitude, and extends research on gratitude by situating it in the underexplored context of a community orchestra. The orchestra members had benevolent intentions to benefit each other. Watkins et al. (2009) argue that gratitude supports happiness because people acknowledge the benefits they receive from others. This was evident in our study: orchestra members valued the beauty of the sound, the kindness of the community, the support and closeness of teamwork, good relationships, opportunities and accomplishments, and the resulting sense of well-being and spiritual enrichment. When participants construed benefactors’ intentions as benevolent, their positive affect increased. All the stakeholders in the orchestra community are benefactors.

Interestingly, music itself was often described as a benefactor, highlighting that in some instances, the benefactor is not human. This aligns with a dyadic or intrapersonal view of gratitude, saying that one can be grateful for something without an implied benefactor (Gulliford et al., 2013). In the orchestra, the aesthetic quality of music is appreciated. Watkins et al. (2009) further link gratitude to aesthetic emotions, a connection supported here by participants’ descriptions of beauty, awe, and admiration for fellow musicians’ passion and talent.

Gratitude also strengthens social relationships (Watkins et al., 2009) and can be considered a social emotion (Roberts, 2004). Participants were grateful for belonging, and our findings illustrate how appreciation chains fuel reciprocity chains, confirming Watkins’s (2004) “cycle of virtue” (p. 184). In Van der Merwe and Morelli’s (2022) relational theoretical framework of how community engagement facilitates social cohesion, belonging is central in motivating participants to return week after week.

Our findings show that musicking creates a shared environment that embraces diverse cultural and generational expressions of gratitude, fostering a cohesive community. Musicking can even promote intercultural understanding (Jones, 2010). Culturally, gratitude is a spiritual practice, often linked to religious and community traditions (McCullough et al., 2002). Younger participants tend to express gratitude more openly and emotionally in interpersonal contexts, while older participants’ expressions of gratitude deepen over time, shaped by an awareness of their experience and the orchestra’s influence. Similarly, Chopik et al. (2019) found that experiences of gratitude were greatest in older adults.

As a moral emotion (Watkins et al., 2009), gratitude motivated participants to contribute to the orchestra’s future. Rituals of gratitude reinforced prosocial behaviour, cohesion, and community. Understanding how appreciation and reciprocity cycles function in ensembles can inform practice. Conductors might implement gratitude rituals, such as reflective prompts during rehearsals or end-of-year acknowledgements, to cultivate positive emotions, strengthen community, and enhance performance.

Since gratitude promotes a positive memory bias (Watkins et al., 2009), targeted rituals may help musicians recall affirming experiences without overuse, which risks diminishing impact. This aligns with Fredrickson’s (2001) broaden-and-build theory, which emphasises how gratitude, as a positive emotion (Fredrickson, 2004), expands awareness and builds enduring resources. Within a community orchestra, gratitude can be understood as broadening mutual attentiveness while building musical skills that enhance performance quality (Bernabé-Valero et al., 2019).

Musicians who demonstrate greater appreciation for positive musical and social experiences tend to show increased resilience in responding to rehearsal and performance challenges, which is associated with higher levels of personal and musical fulfilment (Bernhard, 2022). Small (1998) argues that the meaning of musicking is grounded in the relationships developed among participants, positioning gratitude as an affective means through which these interpersonal connections are supported and nurtured. Musical performance unfolds in real time and depends on continuous responsiveness to others, thereby creating frequent opportunities for mutual support and appreciation. In this way, gratitude is embedded in the practice of participation and performance, emerging through sustained collaborative engagement.

Gratitude in a community orchestra emerges as an aesthetic, social, and moral emotion, virtue and action. By intentionally nurturing gratitude, ensembles can foster upward spirals of appreciation, reciprocity and broadened thought and action cycles that deepen belonging, promote flourishing, and enrich musical achievement.

## Data Availability

The raw data supporting the conclusions of this article will be made available by the authors, without undue reservation.
