# Supplementary material for: Exploring experiences of gratitude during musicking in a community orchestra: an intrinsic case study
Source: Front Psychol. 2026 Mar 6;17:1717657. doi: 10.3389/fpsyg.2026.1717657 (PMC13002394; doi:10.3389/fpsyg.2026.1717657)
Supplement: Supplementary file 1 [file Data_Sheet_1.docx]

**Interview questions:**

**Exploring experiences of gratitude during musicking in a community orchestra: An intrinsic case study**

**Orchestra members/ *Orkeslede***

1. Demographic questions/ *Demografiese vrae*
2. What is your name and surname?/ *Wat is jou naam en van?*
3. What do you want your pseudonym to be?/ *Wat wil jy hê moet jou skuilnaam wees?*
4. What is your age?/ *Hoe oud is jy?*
5. What is your ethnic/cultural background? What is your mother tongue?/ *Wat is jou etniese of kulturele agtergrond? Wat is jou moedertaal?*
6. Tell me about your musical development./ *Vertel my van jou musikale ontwikkeling.*
7. What instrument do you play in the orchestra?/ *Watter instrument speel jy in die orkes?*
8. What position do you play in the orchestra?/ *Watter posisie het jy in die orkes?*
9. How old were you when you started with lessons?/ *Hoe oud was jy toe jy begin les neem het?*
10. What age were you when you joined the orchestra?/ *Hoe oud was jy toe jy by die orkes aangesluit het?*
11. What is your motivation for learning to play this instrument?/ *Wat motiveer jou om hierdie instrument te leer speel?*
12. Tell me about your participation in the orchestra./ *Vertel my van jou deelname in die orkes.*
13. What motivated you to play in the orchestra?/ *Wat motiveer jou om in die orkes te speel?*
14. What do you expect to gain from your orchestral experience?/ *Wat verwag jy om uit jou orkeservaring te kry?*
15. What is your favourite part of playing in the orchestra?/ *Wat is jou gunsteling deel van om in die orkes te speel?*
16. What is your least favourite part of playing in the orchestra?/ *Waarvan hou jy die minste van om in die orkes te speel?*
17. What are your most positive memories of the orchestra so far?/ *Wat is jou mees positiewe herinneringe van die orkes tot dusvêr?*
18. Tell me about your relationships with the orchestra conductors./ *Vertel my van jou verhoudinge met die orkes dirigente.*
19. Tell me about your relationships with your fellow orchestra members./ *Vertel my van jou verhoudinge met jou mede orkeslede.*
20. Tell me about how you envision your future in the orchestra./ *Hoe sien jy jou toekoms in die orkes?*
21. Tell me about your general feelings of gratitude./ *Vertel my van jou algemene gevoel van dankbaarheid.*

*(From: The Gratitude Questionnaire-Six-Item Form (GQ-6) is a six-item self-report questionnaire designed to assess individual differences in the proneness to experience gratitude in daily life. McCullough, M. E., Emmons, R. A., & Tsang, J. (2002). The grateful disposition: A conceptual and empirical topography. Journal of Personality and Social Psychology, 82, 112-127.)*

How do you feel about the following statements, do you agree with it or do you disagree with it? Could you say why you agree or disagree with the statements? / *Hoe voel jy oor die volgende stellings, stem jy saam daarmee of verskil jy daarmee? Kan jy verduidelik hoekom jy met die stellings saamstem of verskil?*

1. I have so much in life to be thankful for./ *Ek het soveel in my lewe om voor dankbaar te wees.*
2. If I had to list everything that I felt grateful for, it would be a very long list./ *As ek ‘n lys sou moes maak met alles waarvoor ek dankbaar is, sou dit ‘n baie lang lys wees.*
3. When I look at the world, I don’t see much to be grateful for./ *Wanneer ek na die wêreld kyk sien ek nie veel om voor dankbaar te wees nie.*
4. I am grateful to a wide variety of people./ *Ek is dankbaar vir ‘n wye verskeidenheid mense.*
5. As I get older, I find myself more able to appreciate the people, events, and

situations that have been part of my life history./ *Soos wat ek ouer word waardeer ek al hoe meer die mense, gebeurtenisse en situasies wat deel van my lewensgeskiedenis was.*

1. Long amounts of time can go by before I feel grateful towards something or

someone./ *Lang tye kan verbygaan voordat ek dankbaar voel teenoor iets of iemand.*

1. Tell me about your experiences of gratitude in the orchestra./ *Vertel my van jou ervaringe van dankbaarheid in die orkes.*
2. Have you ever felt gratitude for the opportunity to be part of the orchestra?/ *Het jy al ooit dankbaar gevoel vir die geleentheid om deel van die orkes te wees?*

If so, what or whom were you grateful towards?/ *Indien wel, waarvoor of teenoor wie was jy dankbaar?*

1. Can you recall a specific moment during orchestra rehearsals or performances when you felt a strong sense of gratitude?/ *Kan jy ‘n spesifieke oomblik gedurende ‘n orkesoefening of konsert onthou wat jy ‘n sterk gevoel van dankbaarheid ervaar het?*

If so, what were the circumstances surrounding that moment?/ *Indien wel, wat was die omstandighede romdom daardie oomblik?*

1. Have you ever received acts of kindness from your fellow orchestra members?/ *Het jy al ooit dade van vriendelikheid van jou mede-orkeslede ontvang?*
2. How has participating in the orchestra influenced your understanding and expression of gratitude toward your fellow musicians?/ *Hoe het deelname aan die orkes jou begrip en uitdrukking van dankbaarheid teenoor jou medemusikante beïnvloed?*
3. Have you noticed any differences in the expression or experience of gratitude based on age differences within the orchestra?/ *Het jy enige verskille opgemerk in die uitdrukking of ervaring van dankbaarheid op grond van ouderdomsverskille binne die orkes?*

If so, how would you describe these differences?/ *Indien wel, hoe sal jy hierdie verskille beskryf?*

1. Have you noticed any cultural differences in the expression or experience of gratitude within the orchestra?/ *Het jy enige kulturele verskille opgemerk in die uitdrukking of ervaring van dankbaarheid binne die orkes?*

If so, how would you describe these differences?/ *Indien wel, hoe sal jy hierdie verskille beskryf?*

1. Can you describe any rituals or traditions within the orchestra that foster a sense of gratitude among members?/ *Kan jy enige rituele of tradisies binne die orkes beskryf wat ‘n gevoel van dankbaarheid onder lede bevorder?*
2. How do you think the experience of gratitude during musicking contributes to the sense of community and belonging within the orchestra?/ *Hoe dink jy dra die ervaring van dankbaarheid tydens musiekmaak by tot ‘n gevoel van gemeenskap en om te behoort binne die orkes?*
3. Have your feelings of gratitude remained the same, increased, or decreased since joining the orchestra?/ *Het jou gevoelens van dankbaarheid dieselfde gebly, toegeneem of afgeneem sedert jy by die orkes aangesluit het?*

Please explain your response./ *Verduidelik asseblief jou antwoord.*

1. Have you ever encountered challenges or obstacles in expressing gratitude towards your fellow musicians in the orchestra?*/ Het jy al ooit uitdagings of struikelblokke teëgekom om dankbaarheid teenoor jou mede-musikante in die orkes uit te spreek?*

If so, how did you manage or overcome these challenges?/ *Indien wel, hoe het jy hierdie uitdagings bestuur of oorkom?*

1. Have you received any support from your family members for playing in the orchestra?/ *Het jy enige ondersteuning van jou familielede ontvang om in die orkes te speel?*
2. How do you think expressing gratitude at the orchestra can benefit your well-being?/ *Hoe dink jy kan die uitdrukking van dankbaarheid by die orkes jou welstand bevoordeel?*
